# Visual Feedback Effectiveness in Reducing Over Speeding of Moped-Riders

**DOI:** 10.3389/fpsyg.2021.611603

**Published:** 2021-03-11

**Authors:** Mariaelena Tagliabue, Riccardo Rossi, Massimiliano Gastaldi, Giulia De Cet, Francesca Freuli, Federico Orsini, Leandro L. Di Stasi, Giulio Vidotto

**Affiliations:** ^1^Department of General Psychology, University of Padua, Padua, Italy; ^2^Department of Civil, Environmental and Architectural Engineering, University of Padua, Padua, Italy; ^3^Mind, Brain, and Behavior Research Center, University of Granada, Granada, Spain

**Keywords:** moped-riding simulator, alert feedback, active warning systems, two-wheel drivers, long lasting learning effects

## Abstract

The use of assistance systems aimed at reducing road fatalities is spreading, especially for car drivers, but less effort has been devoted to developing and testing similar systems for powered two-wheelers (PTWs). Considering that over speeding represents one of the main causal factors in road crashes and that riders are more vulnerable than drivers, in the present study we investigated the effectiveness of an assistance system which signaled speed limit violations during a simulated moped-driving task, in optimal and poor visibility conditions. Participants performed four conditions of simulated riding: one baseline condition without Feedback, one Fog condition in which visual feedback was provided so as to indicate to the participants when a speed limit (lower than that indicated by the traffic signals) was exceeded, and two post-Feedback conditions with and without Fog, respectively, in which no feedback was delivered. Results showed that participants make fewer speeding violations when the feedback is not provided, after 1 month, and regardless of the visibility condition. Finally, the feedback has been proven effective in reducing speed violations in participants with an aggressive riding style, as measured in the baseline session.

## Introduction

Currently, a lot of attention is being paid to the causal role of risky driving behaviors in road crash occurrence, in the context of the efforts dedicated, in the last decades, to the reduction of road fatalities worldwide. Indeed, over speeding remains one of the main causes (among others) of road accidents. In all the countries involved in the Road Safety Annual Report 2019 (OECD/ITF, 2019), speeding seemed to contribute from 15% up to 35% of fatal crashes in 2018. In Italy, according to the Italian National Institute of Statistics, speeding caused 10.2% of road accidents in 2018 and over speeding turned out to be the main contributing factor in 10.3% of injury crashes and 18.5% of fatal crashes in 2017 (OECD/ITF, 2019-Italy).

Speed choice is one of the aspects of driving behavior under the control of drivers, and over speeding represents a risky behavior that can be influenced in several ways. For instance, Lucidi et al. (2019a) recommended to focus educational interventions on drivers’ attitudes toward traffic safety, which can be modified to a greater extent than other aspects that have been proven to influence driving behaviors. Indeed, starting from the “personality-attitudes” model of Ulleberg and Rundmo (2003), Lucidi et al. (2019b) recently confirmed that personality characteristics influence actions, i.e., driving aberrant behaviors, both directly and indirectly through attitudes. In their studies, they replicated a previous finding of different subtypes of drivers, based on personality traits (Gianfranchi et al., 2017a), either in adolescent moped drivers (Lucidi et al., 2019b) or in young, adult, and old car drivers (Lucidi et al., 2019a). The authors, reasoning that attitudes are less stable than personality traits, concluded that interventions targeted on attitudes should be considered the most effective approach (Lucidi et al., 2019a).

Another way to try to reduce risky behaviors such as over speeding, is by acting directly on the actions that need to be corrected, by means of driving assistance systems which provide on-line feedback to induce the driver to modify her/his behavior (behavioral perspective). Despite the great variety of advanced driving assistance systems developed and tested on cars, less effort has been devoted to powered two-wheeler (PTW) riders (Amback et al., 2009; Beanland et al., 2013; Savino et al., 2019). This is even more surprising, considering that riders are more prone to bad outcomes when involved in a crash and continue to be among the most vulnerable category of road users in 2018, in all the IRTAD countries, except United States (OECD/ITF, 2019).

Considering the causal role of over speeding in road crash occurrence (OECD/ITF, 2019), systems based on speed detection (among others) could be fruitfully applied to PTWs, and virtual reality – i.e., simulated riding—might represents a useful tool for investigations aimed at providing crucial information for the development of these kinds of assistance systems.

### Previous Studies

A review of Bayly et al. (2006) showed that among 35 existing in-vehicle systems created to improve road safety, only six are directly implemented in motorcycles. These systems are crucial to shifting from a protection (in case of collision) point of view to a collision prevention perspective, i.e., from a reactive to a proactive approach to road safety.

Moreover, the role of feedback in reducing crash risk is crucial, not only as to the immediate information provided to the driver in risky situations but also as to allow him/her to avoid crashing; indeed studies also demonstrated that some feedback systems lead the driver to behave in a safer way, so as to reduce the likelihood that a risk develops. These behavioral changes may still be evident 1 month later, even if the feedback is no more presented (Rossi et al., 2020).

These studies are often carried out by means of driving simulators which allow drivers to be exposed to hazardous situations in a safe context, and to test the persistence over time of the benefits acquired, demonstrating that the effects of such training may be evident 1 year after the training and are influenced, in turn, by the on-road experience (Vidotto et al., 2015). Furthermore, simulators are the key tools for properly investigating potentialities of in-vehicle systems, since they allow all the variables involved to be manipulated, so as to realize fine-graded testing of the prototypes and to show which setting provides better effects (Rossi et al., 2017; Biondi et al., 2020).

On the basis of the previous considerations, research aimed at improving safety in motorcyclists should follow two main directions: to identify the information, related to the motorcyclist behavior, which can be considered as signals of potential risky driving; and to deeply investigate the ways in which this information can be used to induce the driver to adopt safer riding behaviors and to provide the know-how about the standards needed for the optimal implementation of these technologies for motorcycles (Amback et al., 2009).

### Aims of the Present Study

For the above mentioned reasons, the aim of the present study was to investigate (a) the effectiveness of an alert system which provides an on-line feedback on over speeding during a simulated moped-riding task, and (b) the persistence of its effect in a one-month period. We started by the consideration of the role that speed control plays in crash prevention. However, we did not focus on aspects related to the development of automatic speed control systems, but we decided to investigate how motorcyclists may be trained to adopt voluntary self-control strategies of speed regulation and reduction, also examining the persistence of the effects recorded over time. To this aim, we realized virtual moped-riding conditions, by means of the Honda Riding Trainer (HRT) simulator, in which the system provided a visual feedback about over speeding to the motorcyclists. In particular, the feedback consisted of a red horizontal rectangle which appeared when a speed limit was exceeded. We wanted to test the different impact of this feedback procedure depending on the baseline riding style of the participants. Moreover, we considered another important aspect, i.e., the optimal position in which such kind of feedback should be shown so as to maximize its effectiveness. As to the latter aspect, we selected three locations that should be usually monitored during a riding task: the speedometer and the two side-view mirrors, so as to avoid unnecessary interferences with the usual visual scanning behavior of the participants.

### Research Questions and Hypotheses

First, we hypothesized that the visual feedback was effective in reducing driving speed. The second hypothesis was that the magnitude of the effect would be greater in participants with a risky baseline driving style. Third, we expected that the effects of the feedback procedure would have been evident after 1 month too, when the feedback was no longer available. Finally, as to the feedback position, we expected to observe effects due to attentive processes. Indeed, if attentional mechanisms would be crucial for feedback processing, the feedback should be better detected when presented in a central position (which does not require lateral shifts of attention), since studies demonstrated different trends, in learning to prevent risk during moped riding, when the potential risk appears straight ahead than when it appears in lateral positions (Tagliabue et al., 2013). Moreover, it is known that horizontal attentional shifting is asymmetrical, with an advantage of shifts toward the right visual hemi-field, due to the lateralization of the attentional brain control system (Spironelli et al., 2006, 2009). Thus we could also expect greater effects for the right than for the left feedback position.

## Materials and Methods

### Participants

Seventy-five voluntary participants (38 females, mean age 24.15; 8,792 annual mileage) were recruited, all with normal or corrected to normal vision, at least 1 year of driving experience (1,000 km annual mileage), and no previous experience with riding or driving simulators. The sample was selected through Facebook advertisements. All the participants were naïve as to the aim of the study and were paid for participating in the experiment.

### Material

The experiment was carried out in the HRT Laboratory of the Department of General Psychology, University of Padua, using the Honda Riding Trainer (HRT) simulator.

**Figure 1:**
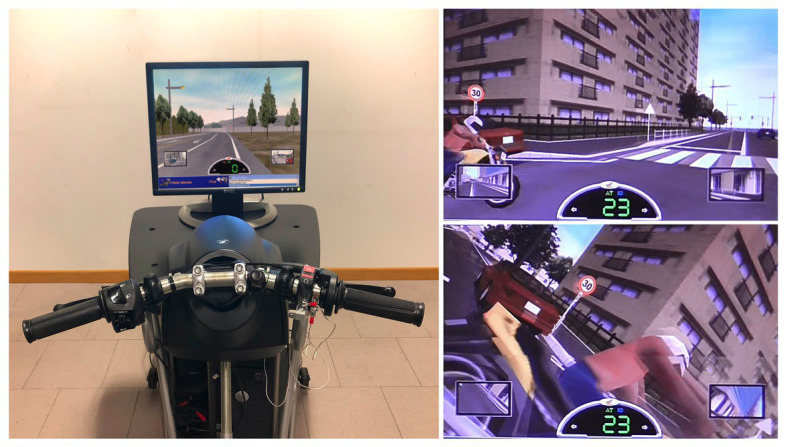
The HRT simulator with an example of risky scene before and during the crash.

The HRT simulator is a moped-riding simulator developed for training skills. It has been proved to be effective in training risk perception and anticipation and allowed to record correlations between personality characteristics and decision-making abilities, on the one hand, and the simulated driving performance on the other hand (Gianfranchi et al., 2017a,b, 2018). Links between simulated riding and different degrees of road exposure and experience have been demonstrated too (Symmons and Mulvihill, 2011). The apparatus includes a handlebar (similar to those of real motorcycles), a motorcycle chassis, and the foot clutch pedal, all connected to a Pentium 4 PC and LCD monitor (1024_768 resolution). The view from side mirrors is represented in the screen, and, in addition, the lateral view is allowed by pressing a button. The distance between the rider and the screen was approximately 80 cm (horizontal angle 27.2° and vertical angle 21.7°; see Figure 1).

It includes several scenarios in main (urban with high traffic density) and secondary (urban with medium traffic density) roads, in which typical risky scenes are shown to train riders to address safety unexpected typical potentially hazardous road situations (see Figure 1). The system was set as a moped in an automatic mode.

Driving parameters were recorded in a log file, besides the evaluation obtained by participants in each risky scene, ranging from 1 (completely safe performance) to 4 (crash occurrence).

The visual feedback consisted in a red horizontal rectangle 7.3 cm long (the same length of the side-view mirrors) and 1.8 cm wide, presented in three different positions depending on the group: along the lower border of the left side-view mirror, of the speedometer, and of the right side-view mirror, respectively.

### Procedure

Three riding sessions were carried out 1 month apart from each other. In each session, two different routes of main urban roads were presented; thus, participants had to ride throughout six different routes distributed across the three sessions. The six routes were distributed across sessions on the basis of their degree of difficulty (Miceli et al., 2008) so as to make homogeneous the overall difficulty of each session:

Session 1: Participants had to ride along two routes of urban roads (different from each other) in optimal daylight conditions (without fog) and no feedback was delivered (preFeedback/NoFog). They were instructed to ride respecting the limit indicated by the traffic signals, as they usually do while driving on the road.
Session 2: Participants had to ride along two new routes of urban roads (different from each other) in Fog conditions with feedback (Feedback/Fog). Because of the adverse climatic conditions (foggy day), they were instructed to ride respecting a lower speed limit of 30 km/h. In this session, a visual feedback was provided when the limit was exceeded.
Session 3: Participants had to ride along two new different routes of urban roads: one route in Fog conditions (postFeedback/Fog) and one route in optimal daylight conditions (postFeedback/NoFog), both approximately one month later the second session and without any feedback. They were instructed to ride respecting the limit indicated by the traffic signals or the lower speed limit of 30 km/h, depending on the visibility condition. The sequence of the route visibility conditions (with and without fog) was counterbalanced between participants.

In summary, four visibility/feedback conditions were realized: the condition without fog and without feedback in the first session (two different routes); the condition with fog and with feedback in the second session (two routes, different from each other and from the previous ones); and two conditions without feedback in the third session, one route with fog and the other route without fog. Note that in this last session, half participants rode the with-fog route first, and then the without-fog route, whereas, to the other half of the participants, the opposite sequence of tasks was administered.

During the first session, after filling out the written informed consent, participants received the instructions and familiarized with the simulator performing one moped-riding training route of 3 min without other road users or hazardous situations. After the familiarization phase, the first baseline condition was administered, consisting in two routes, each lasting approximately 6–7 min.

**Figure 2:**
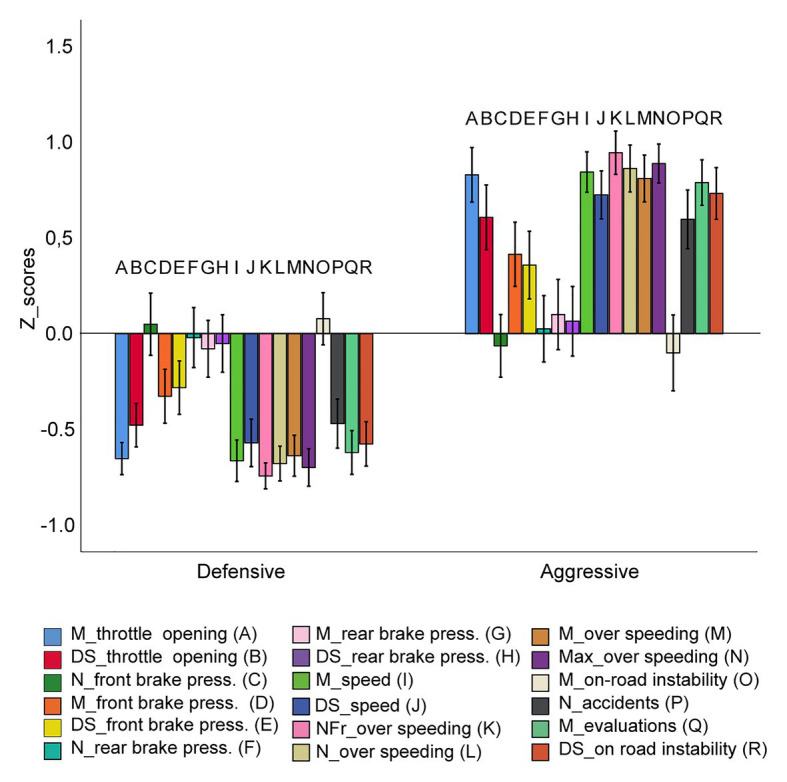
Patterns of Z-scores of the 18 HRT indexes in the two clusters. Legend: mean and standard deviation of the throttle opening (A and B respectively); number, mean, and standard deviation of brakes with the front brake (C, D, E); number, mean, and standard deviation of brakes with the rear brake (F, G, H); mean and standard deviation of speed (I and J); time spent over the speed limit (K); number, mean, and the highest value of over speeding (L, M, N); mean (O) and standard deviation (R) of on-road instability; number of accidents (P) and evaluation score (Q). Vertical bars represent standard errors.

After the first session, two riding style groups—Defensive vs. Aggressive—were identified through a cluster analysis on 18 performance variables extracted from the simulator (see Figure 2 for more details). Then, participants were assigned to three groups of 25 participants, in which gender, riding style, and annual mileage were balanced. Each group was assigned to one of the three experimental conditions, depending on the position of the visual feedback:

Condition a: (12 females—7 with a defensive riding style and 13 males—7 defensive riders). Feedback on the lower edge of the left side-view mirror.
Condition b: (13 females—7 with a defensive riding style and 12 males—7 defensive riders). Feedback on the lower edge of the speedometer.
Condition c: (13 females—8 with a defensive riding style and 12 males—6 defensive riders). Feedback on the lower edge of the right side-view mirror (means and standard deviations of age for each group are reported in Table 1).

**Table 1:** Means and standard deviations of age for each group.

Riding style groups	Feedback position	Mean age	St. dev.
Defensive	Left	23.86	2.96
	Center	24.79	3.21
	Right	24.50	2.44
Aggressive	Left	23.82	4.07
	Center	23.91	1.51
	Right	23.82	2.40

The study was approved by the Ethical Committee for the Psychological Research of the University of Padua (protocol N° 3259, code 4E55A90D9EACFBA2EE569B18AE7CACDF, 11/20/2019).

### Experimental Design and Data Analyses

All the analyses were carried out with IBM SPSS 22 statistical package. After the first session, a hierarchical cluster analysis, using the Ward’s method with squared Euclidean distance, was performed on the 18 riding parameters of each participant. This preliminary analysis showed the presence of two clusters. Then, on the Z-scores of the driving parameters we applied a K-means clustering method, so as to extract the best clustering solution.

To test our specific hypotheses, we carried out two ANOVAs on the percentage of over speeding with reference to the 30-km/h limit and to the limits indicated by the traffic signals, respectively, both with two between-participant factors, i.e., Cluster (2 levels: Defensive vs. Aggressive) and Feedback position (three levels: left, center, and right), and Visibility condition (four levels: preFeedback/NoFog, Feedback/Fog, postFeedback/Fog, and postFeedback/NoFog) as the within-participant factor.

## Results

### Cluster Solution

The final solution of the cluster analysis showed the presence of two clusters with different riding patterns. In the first cluster, named “Defensive,” 42 participants were included (22 females and 20 males; annual mileage 8,257) whereas 33 participants were clustered in the second riding style group named “Aggressive” (16 females and 17 males; annual mileage 9,473). In Figure 2, the mean Z-scores of the HRT indexes for the two clusters are shown. As it can be seen, the Defensive cluster shows overall better performance evaluations, supported by lower number of accidents, speed, and acceleration rate, than the Aggressive cluster.

### Analyses of Over Speeding

Two participants were discarded since they withdrew before the third session (1 female—Defensive, Condition b and 1 male—Aggressive, Condition a).

In the first ANOVA on the percentages of breaking the 30 km/h speed limit, the factors Cluster and Visibility condition reached significance with *F*(1,67) = 60.60, *p* < 0.001, *η*_p_^2^ = 0.47 and *F*(3,201) = 110.93, *p* < 0.001, *η*_p_^2^ = 0.62, respectively. Aggressive riders showed higher percentage of over speeding than Defensive riders (12.89% vs. 23.70%). Moreover, in the preFeedback/NoFog condition the percentage of over speeding was higher (30.4%) compared with those of the other three visibility conditions, and in the two Fog visibility conditions, the percentages of over speeding were similar (9.4 and 12.2%) and lower than that in the postFeedback/NoFog condition (21.1%). No other source of variance or interaction reached significance.

**Figure 3:**
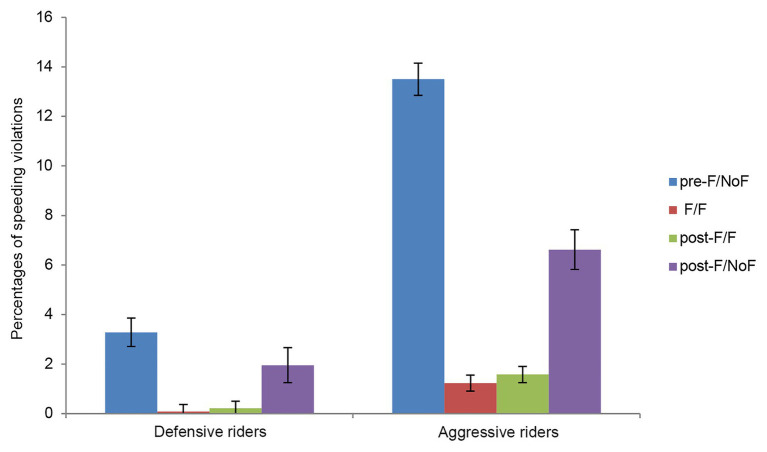
Percentage of speeding violations (over the limits indicated by traffic signals) of Defensive and Aggressive riders in the four visibility conditions. Vertical bars represent standard errors.

In the second ANOVA on the percentage of violations of the speed limit as indicated by the traffic signals, the factors Cluster, Visibility condition and the interaction Cluster X Visibility condition reached significance with *F*(1,67) = 74.66, *p* < 0.001, *η*_p_^2^ = 0.53, *F*(3,201) = 125.44, *p* < 0.001, *η*_p_^2^ = 0.65, and *F*(3,201) = 42.76, *p* < 0.001, *η*_p_^2^ = 0.39, respectively. Defensive riders made fewer speeding violations than Aggressive riders (1.38% vs. 5.73%). In the fog conditions, participants made the same percentage of speeding violations (0.66 vs. 0.90), but fewer speeding violations than in the NoFog conditions, in which violations were fewer in the post-F condition (4.29% vs. 8.39%). Finally, the interaction showed that the reduction in the percentage of speeding violations in the postFeedback/NoFog condition is significant only in the Aggressive rider group (see Figure 3). No other source of variance or interaction reached significance.

## Discussion

The results of the analysis on the lower speed limit violations showed that the feedback appears to be effective in reducing speed under poor (and partially under optimal) visibility conditions after one month, indicating that it induces a more prudent riding behavior, not only when it is delivered but also when it is no more provided. This seems to indicate a strong effectiveness of the feedback, since the behavior modifications observed are quickly acquired in a 12–15-min session and persist over time, so as to manifest also when visibility conditions are optimal.

As to the effect of feedback position, it seems not to influence performance. On the basis of the knowledge about the mechanisms which control attentive shifting, we expected a different degree of feedback effectiveness dependently on the feedback position. The fact that this prediction has not been confirmed seems to indicate that the processes underlying feedback detection do not increase the attentional demand of the task. This may be due to the particular positions chosen: all the three positions are typically salient while riding. In other words, riding (as driving in general) requires always to monitor the speedometer and the side-view mirrors. Further research in which feedback appears in other less crucial positions may help in disentangling this matter.

## Conclusion

The present study, focused on the effectiveness of visual feedback in reducing risky behaviors such as over speeding, has shown that a simple visual feedback that indicates, in a discrete way (differently from the speedometer that provides a continuous flow of information), when a certain speed threshold is exceeded, induces in a very short time behavioral modifications that tend to persist over time.

Moreover, it also seems to induce safer behaviors, with regard to the traffic rules, in riders with an aggressive riding style. In other words, feedback systems that induce riders to reduce speed in low-visibility conditions make Aggressive riders less prone toward speeding violations (to exceed less frequently the legal limit) even in optimal visibility conditions, and even when the feedback is removed.

One limit might rely on the interpretation of the effects of the feedback. Indeed, it can be argued that the more cautious riding behavior observed in the last session, when the feedback is not delivered and the visibility is good, might be due to the fact that participants have developed better speed-control strategies because of their expectations or beliefs about experimental goals. Note that this effect should not be considered as a contextual learning effect related to the specific characteristics of the routes, since the administered routes were different in all the conditions. Conversely, participants could have learned that their speed was monitored. However, this alternative explanation, which surely may be considered with regard to the last session in poor visibility conditions, seems not so compelling for the no-fog condition, since in no session were participants required to respect a speed limit lower than that indicated by the traffic signals, in optimal daylight conditions.

Testing of the duration over longer periods of time of the effects of the feedback should be considered too, since it can provide intuitions about the way in which assistant systems may be more successfully used and it can suggest important insights for educational interventions aimed at promoting safe driving.

In the present study, driving simulation has proved to be an efficient instrument for providing information to the developer of assistance systems able to improve road safety in PTW-riders.

As a final remark, it is worthy to emphasize, in line with the recommendation of the European Commission (2018), that to tackle problems related to road safety effectively, multiple approaches should be employed. As previously highlighted, educational programs aimed at reducing aberrant driving behavior should be developed in different directions: from the intervention on attitudes toward traffic safety (Lucidi et al., 2019a) to the use of trainings pointing to directly modify behaviors, also through the use of in-vehicle systems which support the driver in speed management.

## Data Availability Statement

The raw data supporting the conclusions of this article will be made available by the authors, without undue reservation.

## Ethics Statement

The studies involving human participants were reviewed and approved by the Ethical Committee for the Psychological Research of the University of Padua. The patients/participants provided their written informed consent to participate in this study.

## Author Contributions

The authors confirm contribution to the paper as follows: RR, MT, MG, LLDS, and GV: study conception and design. GDC, FF, and FO: data collection. RR, MT, MG, GDC, FF, FO, LLDS, and GV: analysis and interpretation of results. RR, MT, MG, GDC, FF, FO, LLDS, and GV: draft manuscript preparation. All authors contributed to the article and approved the submitted version.

### Conflict of Interest

The authors declare that the research was conducted in the absence of any commercial or financial relationships that could be construed as a potential conflict of interest.
